# Midkine, heparin-binding growth factor, blocks kainic acid-induced seizure and neuronal cell death in mouse hippocampus

**DOI:** 10.1186/1471-2202-11-42

**Published:** 2010-03-26

**Authors:** Yun B Kim, Jae K Ryu, Hong J Lee, In J Lim, Dongsun Park, Min C Lee, Seung U Kim

**Affiliations:** 1 Division of Neurology, Department of Medicine, UBC Hospital, University of British Columbia, Vancouver, Canada; 2 College of Veterinary Medicine, Chungbuk National University, Cheongju, Korea; 3 Medical Research Institute, Chung-Ang University College of Medicine, Seoul, Korea; 4 Department of Physiology, Chung-Ang University College of Medicine, Seoul, Korea; 5 Department of Pathology, Chonnam National University Medical School, Gwangju, Korea

## Abstract

**Background:**

Midkine (MK), a member of the heparin-binding growth factor family, which includes MK and pleiotrophin, is known to possess neurotrophic and neuroprotective properties in the central nervous system. Previous studies have shown that MK is an effective neuroprotective agent in reducing retinal degeneration caused by excessive light and decreasing hippocampal neuronal death in ischemic gerbil brain. The present study was undertaken to investigate whether MK acts as an anticonvulsant in kainic acid (KA)-induced seizure in mouse and blocks KA-mediated neuronal cell death in hippocampus.

**Results:**

Increased expression of MK was found in hippocampus of mouse following seizures induced by intracerebroventricular injection of KA, and MK expression was found in glial fibrillary acidic protein (GFAP)-positive astrocytes. Concurrent injection of MK and KA attenuated KA-induced seizure activity and cell death of hippocampal neurons including pyramidal cells and glutamic acid decarboxylase 67 (GAD67)-positive GABAergic interneurons in the CA3 and hilar area.

**Conclusion:**

The results of the present study indicate that MK functions as an anticonvulsant and neuroprotective agent in hippocampus during KA-induced seizures.

## Background

Temporal lobe epilepsy (TLE) is pathologically characterized by extensive neuronal loss in the CA1, CA3 and hilar regions of hippocampus [1,2]. Previous studies have demonstrated that the animal models of TLE generated by intracerebroventricular injection of kainic acid (KA) faithfully reproduce clinical and pathological features found in human TLE [3-7].

Previous studies have reported the possible involvement of neurotrophic factors in epilepsy as suggested by the gene expression of neurotrophic factors such as NGF, BDNF and NT-3 in hippocampus in human TLE as well as in TLE animal models [8,9]. Midkine (MK), one of such neurotrophic factors, has emerged as an important neuromodulator in the central nervous system (CNS). MK, a member of the heparin-binding growth factor family, which includes MK and pleiotrophin, is known to possess neurotrophic and neuroprotective properties [10,11]. MK was originally isolated as the product of retinoic acid-responsive gene that functions primarily in inducing cell differentiation in mouse teratocarcinoma cells [12], and has the ability to influence a variety of neuronal functions including neurite extension [13], neuronal differentiation [14,15] and neuronal survival following injury or damage in the CNS [15,16]. During the fetal development of the CNS, MK expression was demonstrated in neuroepithelial/neural progenitor cells following ethylnitrosourea injury [17] indicating that MK might have a role in cellular proliferation [18]. Recent studies further showed that MK has been implicated in neurological diseases, including Alzheimer's disease [19], cerebral ischemia [20] and Parkinson-dementia complex of Guam (Lytico-bodig disease) [21]. In patients with Alzheimer's disease [19] or Lytico-bodig disease [21], MK immunoreactivity was found in senile plaques and neurofibrillary tangles. In addition, an increased expression of MK was found in astrocytes in rat models of cerebral ischemia [22]. It is not known, however, whether the expression of MK in the brain after the brain injury is a part of an endogenous repair process to prevent further damage in the CNS.

The objectives of the present study are to determine whether intracerebroventricularly injected MK acts as an anticonvulsant and blocks KA-mediated neuronal cell death in hippocampus.

## Results

### MK expression after seizures

**Figure 1:**
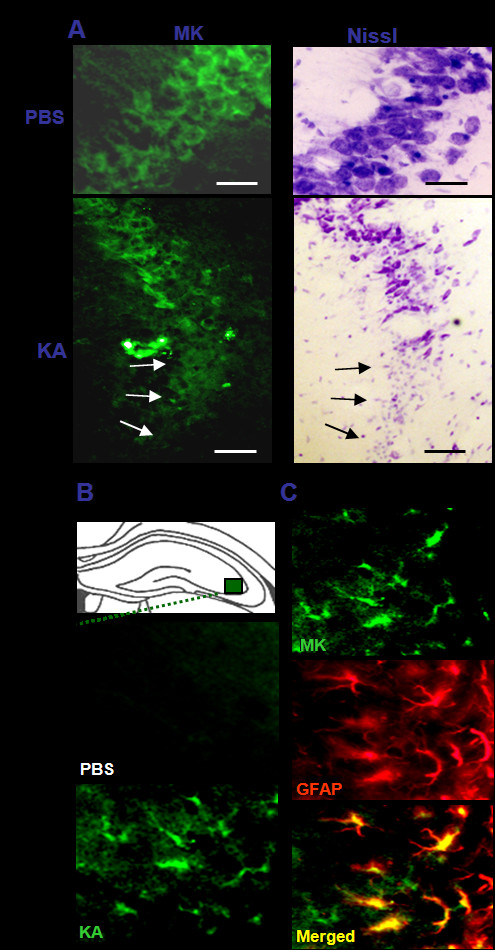
**MK expression in the hippocampus after KA injection**. (A) Representative immunofluorescence images of MK immunoreactivity in hippocampal CA3 pyramidal neurons 24 hr after PBS injection (top left panel) or KA (0.2 μg/mouse, bottom left panel). Adjacent hippocampal sections stained with Nissl staining (right panels) are shown here. Following KA treatment, cell death in CA3 pyramidal neurons is clearly visible (arrows). Scale bars: 20 μm (top panels), 30 μm (bottom panels). (B) Representative immunofluorescence images of MK-positive cells in CA3 Slm (striatum lacunosum moleculare) (box in top panel indicates region of interest) after injection with PBS (middle panel) or KA (0.2 μg/mouse, bottom panel). (C) Double-labeling immunofluorescence staining of MK (green, top panel) with GFAP (red, middle panel) showing MK immunoreactivity is co-localized on GFAP-positive astrocytes in CA3 Slm (bottom panel). Scale bar: 20 μm.

We first examined MK expression immunohistochemically in mouse hippocampus following KA injection. Injection of KA (0.2 μg/mouse) to mice induced severe epileptiform seizures (mean score 4.2/maximum score 5.0). Basal level of MK immunoreactivity was found in hippocampal pyramidal neurons in control mouse brain injected with vehicle [phosphate-buffered saline (PBS)] (Figure 1A, top left panel), while in mouse injected with KA decreased MK expression was detected in pyramidal neurons (Figure 1A, bottom left panel; see arrows); Nissl staining of the adjacent sections confirmed that the cellular area of decreased MK immunoreactivity was associated with damaged pyramidal neurons (Figure 1A, bottom right panel; see arrows). Nissl staining in control animals receiving PBS injection showed no evident neuronal damage (Figure 1A, top right panel).

Interestingly, we have found the number of MK-positive cells markedly increased in stratum lacunosum moleculare (Slm) area of CA3 at 24-hr post-KA injection relative to PBS controls. Representative MK immunostaining in CA3 Slm is shown in Figure 1B (box denotes area of high magnification, top panel). In PBS-injected brain, only small number of MK-positive cells was observed (Figure 1B, middle panel). Following injection of KA, CA3 Slm area demonstrated an increase in the number of MK-positive cells (Figure 1B, bottom panel). Double-labeling immunofluorescence staining was then used to investigate MK expression in glial cells in KA-injected hippocampus. Representative double staining for MK (green staining, Figure 1C top panel) and GFAP (red staining, Figure 1C middle panel) showed that MK was expressed in GFAP-positive astrocytes; there was no MK expression in anti-complement receptor 3 (OX-42)-positive microglia (data not shown).

**Figure 2:**
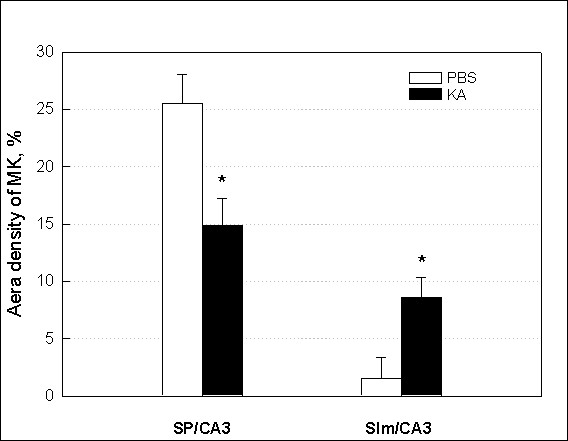
**Site-selective MK expression in the KA-damaged hippocampus**. Bar graph shows the quantification of MK immunoreactivity in SP (stratum pyramidale) and Slm (stratum lacunosum moleculare) of hippocampal CA3 region 24 hr after PBS or KA (0.2 μg/mouse) injection. Data are presented as mean ± SEM. * *p *< 0.05, as compared with PBS. One-way ANOVA, Student-Newman-Keuls multiple comparison test.

Quantification of MK expression data (expressed as area density of MK-positive cells, Figure 2) showed that KA injection significantly decreased MK immunoreactivity by 44% in SP area relative to PBS-injected control. However, the MK immunoreactivity in animals with KA injection markedly increased by 651% in Slm area as compared to PBS-injected control.

### Anticonvulsant effect of MK

To further investigate the role of MK in KA-injected hippocampus, we examined the potential protective effects of MK against KA-induced seizures and neurotoxicity. Seizure onset was approximately 170 sec following KA injection, which lasted for mean 1,200 sec (Table 1). The intensity of seizures reached mean score 4.2 (maximum score 5.0) when the seizure activity was highest (5-10 min after KA challenge). Interestingly, seizure duration and intensity were markedly reduced by co-administration of MK in a dose-dependent manner, although the time of onset time slightly delayed at a high dose (0.4 μg/mouse). Seizure duration and intensity were shortened and attenuated to 51.4 - 26.5% and 59.5 - 40.5% of the control levels by treatment with KA (0.1 - 0.4 μg/mouse), respectively.

**Table 1:** Effects of MK on KA-induced seizure activities

Treatment (μg/mouse)	Latency to onset (sec)	Seizure duration (sec)	Seizure intensity (maximum score 5.0)
Vehicle	0.0 ± 0.0	0.0 ± 0.0	0.0 ± 0.0
KA (0.2) alone	169.9 ± 38.8*	1,200.6 ± 372.3*	4.2 ± 0.1*
+MK (0.1)	160.0 ± 28.5	616.7 ± 50.3^#^	2.5 ± 0.3^#^
+MK (0.2)	172.8 ± 33.8	422.4 ± 71.5^#^	2.2 ± 0.2^#^
+MK (0.4)	188.8 ± 47.0	318.6 ± 31.7^#^	1.7 ± 0.3^#^

Data are presented as mean ± SEM. * *p *< 0.05, as compared with PBS. # *p *< 0.05, as compared with KA. Student's *t*-test.

### Neuroprotective effect of MK

**Figure 3:**
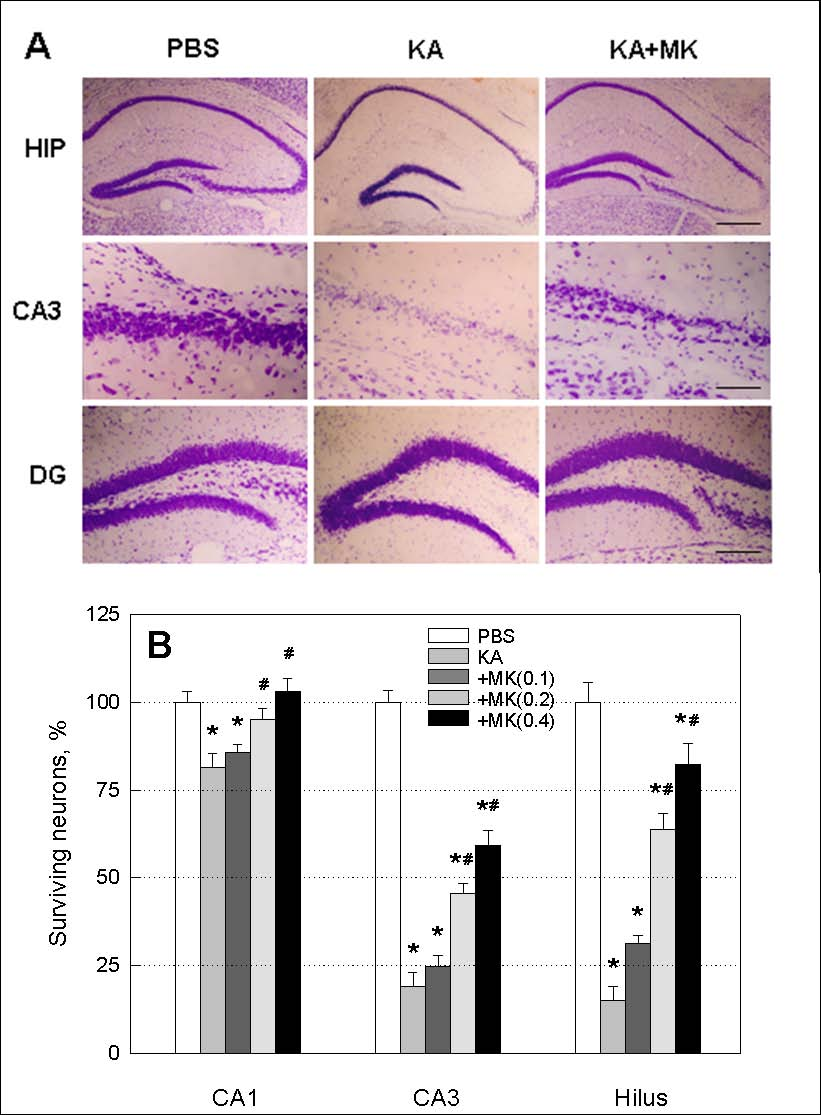
**Effect of MK on hippocampal neuronal damage induced by KA injection**. (A) Representative Nissl-stained sections from animals 24 hr after PBS (left panels), KA (0.2 μg/mouse, middle panels) or KA plus MK (0.4 μg/mouse, right panels) injection. Top panels shows at low magnification of the hippocampus. Scale bar: 400 μm. Middle and bottom panels show at high magnification of CA3 and dentate gyrus (DG) subregions of hippocampus. Scale bars: 200 μm. Note that the damaged areas induced by KA are indicated by absence of Nissl-positive neurons. (B) Quantification of undamaged Nissl-positive neurons for the hippocampal subregions CA1 and CA3, and hilus of dentate gyrus with different MK doses (0.1 -- 0.4 μg/mouse). Data are presented as mean ± SEM. * *p *< 0.05, as compared with PBS. # *p *< 0.05, as compared with KA. One-way ANOVA, Student-Newman-Keuls multiple comparison test.

Representative Nissl staining 24 hr following KA injection showed considerable neuronal loss in the hippocampal subregions, CA3 and hilus of the dentate gyrus as compared to PBS control (Figure 3A, left and middle panels). MK treatment was effective in reducing neuronal loss in KA-injected hippocampus as shown in a representative finding in a high dose (0.4 μg/mouse) group (Figure 3A, right panels). The extent of degeneration of hippocampal neurons was quantified in hippocampal subregions, CA1, CA3, and hilus (Figure 3B). The number of hippocampal neurons was significantly reduced in CA3 (-81%) and hilus (-85%) by KA exposure, whereas CA1 region (-19%) was relatively spared when compared with PBS control. Co-application of MK increased survival of neurons in KA-injected hippocampus in a dose-dependent manner, leading to significant improvements at KA doses of 0.2 and 0.4 μg/mouse.

**Figure 4:**
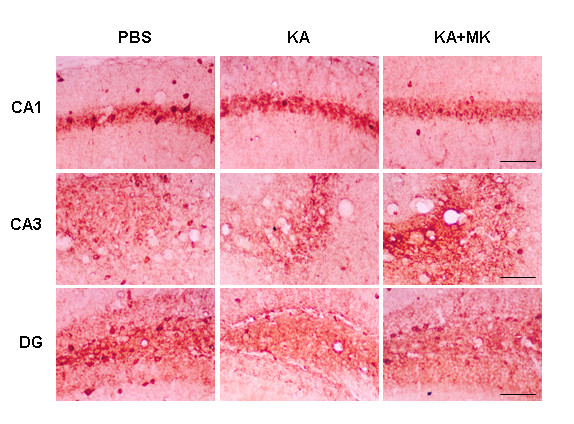
**Protective effect of MK on KA-induced cell loss of GAD67-positive GABAergic neurons**. Hippocampal coronal sections from animals were immunostained with glutamic acid decarboxylase 67 (GAD67) antibody 24 hr after PBS (left panels), KA (0.2 μg/mouse, middle panels), KA plus MK (0.4 μg/mouse, right panels) injection. High magnification of representative GAD67-stained images of hippocampal subregions CA1 (top panels), CA3 (middle panels), and DG (bottom panels) from different treatments are shown here. Scale bar: 200 μm. Note the paucity of GAD67-positve cells in KA-treated CA3 and dentate gyrus subregions.

**Figure 5:**
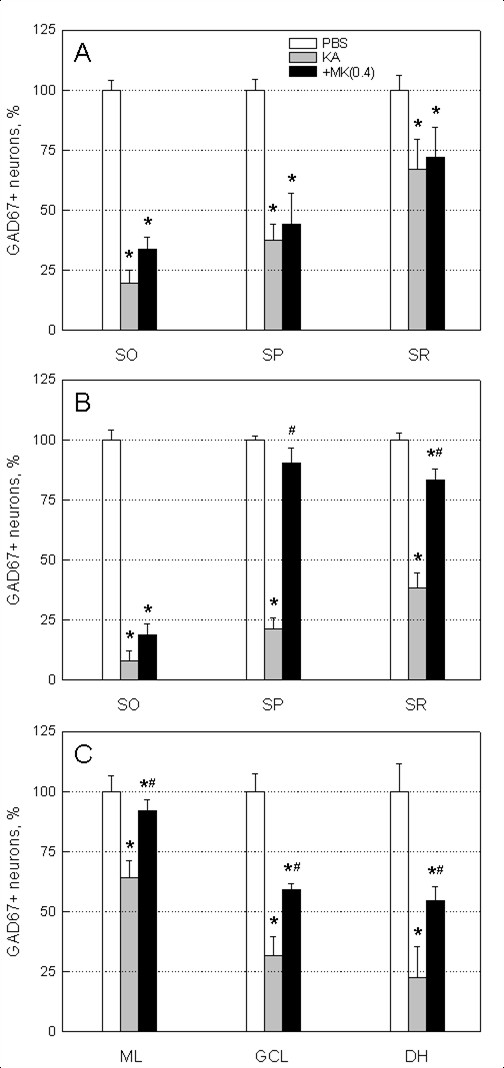
**Protective effect of MK on KA-induced GABAergic neuronal cell death**. Numbers of GAD67+ neurons were counted in subregions of hippocampus from PBS, KA (0.2 μg/mouse), and KA plus MK (0.4 μg/mouse)-injected mice; strata oriens (SO), pyramidale (SP) and radiatum (SR) of CA1 (A) and CA3 (B) and all layers of dentate gyrus (C) (ML; molecular layer, GCL; granule cell layer, DH; dentate hilus). Data are presented as mean ± SEM. * *p *< 0.05, as compared with PBS. # *p *< 0.05, as compared with KA. One-way ANOVA, Student-Newman-Keuls multiple comparison test.

Next we investigated the efficacy of MK to block the KA-induced cell death of GABAergic interneurons (GAD67-positive neurons). A previous study has reported that GAD67-positive interneurons are lost in the hippocampus in KA-injected animal model of excitotoxicity [23]. Animals receiving KA injection showed significant reduction in the number of GAD67-positive interneurons in subfields of CA1 (strata oriens -80%, strata pyramidale -64%, strata radiatum -33%), CA3 (strata oriens -91%, strata pyramidale -78%, strata radiatum -62%) and layers of dentate gyrus (molecular layer -37%, granule cell layer -69%, dentate hilus -77%) as compared to PBS control (Figures 4 and 5). Administration of MK (0.4 μg/mouse) reduced the cell loss in GAD67-positive neurons caused by KA injection and this neuroprotective effect of MK was evident in the subfields of CA3 and layers of dentate gyrus (Figure 4). Application of MK markedly increased survival of GAD67-positive neurons in the strata pyramidale (68% improvement), radiatum (45% improvement) of CA3 and molecular layer (27% improvement), granule cell layer (29% improvement), and dentate hilus (33% improvement) of dentate gyrus, whereas neurons in subfields of CA1 was not effectively preserved (Figure 5).

## Discussion

The main finding of this study is that intracerebroventricular administration of MK conferred neuroprotection against KA-induced excitotoxic cell death of hippocampal neurons. Our results also demonstrated that MK was effective in attenuating KA-induced seizures and degeneration of GABAergic interneurons in hippocampus. The results from immunohistochemical staining showed that damaged pyramidal neurons in hippocampus are correlated with the decreased level of MK expression (Figure 1A). At present, it is not known whether this reduced level of MK immunoreactivity in neurons is causative factor for neuronal degeneration induced by KA injection. However, previous *in vivo *studies have demonstrated that MK is an effective neuroprotective agent in reducing retinal degeneration caused by excessive light [24] and decreasing hippocampal neuronal death in ischemic gerbil brain [25]. Furthermore, MK knock-out mice displayed altered expression of calcium-binding protein in hippocampus and defective working memory [26]. In view of these observations, it is possible that MK is involved in the regulation of endogenous neuroprotective process against externally applied injury or disease.

We have found that the level of MK immunoreactivity markedly increased in astrocytes after KA injection (Figure 1C). In a previous report, we have demonstrated rapid activation of astrocytes in hippocampal alveus and fimbria, strata oriens and lacunosum moleculare as well as dentate hilus as early as 1 hr following diisopropyl fluorophosphate (DFP)-induced seizures and neuronal injury [27]. These observations suggest that the activated astrocytes serve as an important source of MK in response to excitotoxicity. Important findings in the present study are therapeutic and neuroprotective effects of MK on KA-induced neuronal injury in CA3 and dentate hilus of hippocampus (Figure 3). Our results are in good agreement with a previous study of neuroprotective effect of MK in transient forebrain ischemia when given immediately before middle cerebral artery occlusion [25]. Previous studies have demonstrated that KA-injected brain show dramatic reduction in the number of GAD-positive interneurons [7,23,28], even in CA1 statum oriens and alveus where pyramidal neurons are relatively spared [Figures 3-5][29], which can cause abnormal functional inhibition of neuronal circuitry leading to hippocampal hyperexcitability [2,7]. Thus, it is noteworthy that icv application of MK blocks the degeneration of GAD67-positive interneurons, especially in the stratum pyramidale and radiatum of CA3, and the molecular layer, granule cell layer and hilus of dentate gyrus as caused by KA injection (Figures 4 and 5).

The majority of the animals experienced stage 4/5 seizure severity after KA injection. A previous study has demonstrated in KA-injected rat epilepsy model that dizocilpine (MK-801) inhibited hippocampal neuronal loss without blocking seizure development [30]. In addition, several studies in animal model of TLE have also shown that significant neuronal loss is not necessarily a prerequisite for the development of seizures [31,32]. Such results imply that the mechanism of MK action in TLE model is different from that of MK-801, an NMDA antagonist. Our results show that MK-induced neuroprotection against KA toxicity is primarily associated with moderation of seizure activity (Table 1). It is believed that MK plays a role as an anticonvulsant directly (Table 1) or indirectly by preserving an inhibitory amino acid (GABA) system including GAD67-positive interneurons (Figures 4 and 5). Furthermore, it is possible to speculate that MK could be a neurotrophic factor, especially for GAD67-positive interneurons or astrocytes expressing high levels of MK (Figure 1) which may contribute to early cessation of seizure and control of its recurrence.

The exact mechanism of the neuroprotective activity of MK remains to be further clarified but activation of gene product(s) associated with apoptosis by MK may provide some answers. For instance, previous studies have shown that MK inhibits apoptotic process by up-regulation of bcl-2 expression [33] and by inhibition of caspase-3 activation [34]. Thus, it is possible that the MK-mediated neuroprotective mechanism involves activation of signal transduction pathways involved in regulation of apoptotic cell death.

## Conclusions

The results of the present study demonstrate that the administration of MK produces a significant neuroprotective effect against KA-induced neuronal loss in mouse model of epilepsy. Additional studies of MK to elucidate their neuroprotective activity in animal models of brain injury and neurodegeneration such as Parkinson's disease, Huntington's disease, amyotropic lateral sclerosis, stroke or spinal cord injury should prove MK as a member of neurotrophic factors that are valuable in providing effective treatment for patients with various neurological disorders.

## Methods

### Treatment and seizure monitoring

Male C57BL/6 mice (n = 8/group) weighing 25 - 30 g were used for the experiments. The animals were housed in a temperature- and humidity-controlled room that was kept on an alternating 2-hr light/dark schedule. Food and water were available *ad libitum *throughout the experiments. All animal experiments were conducted in accordance with the on Animal Care Committee of the University of British Columbia.

Animals were anesthetized with intraperitoneal injection of chloral hydrate (7%, 0.1 mL/kg) and then mounted in a stereotaxic apparatus (David Kopf Instruments, Tujunga, CA). KA (0.2 μg/0.4 μL PBS/mouse; Sigma, St. Louis, MO) was injected into the right lateral cerebroventricle at the coordinate (AP, -2.0; ML, -2.9; DV, -3.8) using a 10 μL Hamilton syringe fitted with 26G needle at a rate of 0.1 μL/min. The needle was left in place for 5 min. MK (0.1, 0.2 or 0.4 μg/0.4 μL PBS; LG Biotech, Daejon, Korea) was unilaterally co-injected with KA into the right lateral cerebroventricle. For sham-operated animals, the same volume of 0.1 M PBS was stereotaxically injected into the same coordinates described above. Wounds were sutured, and animals were allowed to recover and then returned to their cages. After KA injection, each animal was placed in a Plexiglas cylinder and their seizure behaviors - latency to onset, duration, and intensity - were recorded for a period of 120 min in a blind manner. Seizure intensity was scored with a slight modification from a previous scoring system [35] as followed: stage 1, immobilization and staring; stage 2, head nodding; stage 3, rearing accompanied by forelimb clonus and wet dog shakes; stage 4, falling and wobbling; stage 5, jumping, circling, or rolling. The starting time of head nodding was considered onset time of seizures, because the starting of immobilization and staring behaviors (stage 1) was not clear.

### Immunohistochemistry

The animals were anesthetized and transcardially perfused with 50 mL cold saline followed by 100 mL of 4% paraformaldehyde in 0.1 M phosphate buffer (pH 7.4) 24 hr after KA administration. The brains were removed from the skull and post-fixed in 4% paraformaldehyde for 24 hr, followed with cryoprotection in 30% sucrose in phosphate buffer for 2 days. Serial coronal sections at 30 μm were prepared on a cryostat (CM 1900; Leica, Heerbrugg, Switzerland). Free-floating sections were prepared from the brains of PBS, KA, and KA plus MK-injected mice. For single immunofluorescence staining, brain sections were incubated in PBS containing 5% normal goat serum and 0.2% Triton X-100 for 30 min at room temperature (RT), and then incubated overnight with rabbit anti-MK antibody (1:500, kindly provided by Dr. T. Muramatsu, Nagoya University, Japan). Sections were then incubated with Alexa Fluor 488-conjugated goat anti-rabbit IgG (1:200; Molecular Probes, Eugene, OR) at RT for 2 hr in the dark. Sections were washed in phosphate buffer, mounted on slides. Nissl staining was also performed on slide-mounted brain sections with 0.1% cresyl violet (Sigma) for the evaluation of hippocampal neuronal loss.

For GAD67 immunohistochemical staining, all incubation solutions did not contain Triton X-100. The brain sections were briefly quenched with 3% H_2_O_2 _in PBS for 10 min and incubated with 5% normal goat serum for 30 min. The sections were incubated overnight at 4°C with rabbit anti-GAD67 (1:1000; Chemicon, Temecula, CA). The sections were then incubated for 1 hr with biotinylated anti-rabbit IgG (1:200; Vector, Burlingame, CA), followed by incubation with avidin-biotin complex (1:200, Vector) for 1 hr and then visualized with 0.05% 3,3'-diaminobenzidine (Sigma) and 0.003% H_2_O_2_. Negative control sections were prepared for immunohistochemical staining in an identical manner except the primary antibodies were omitted.

### Double-labeling immunofluorescence microscopy

Free floating sections were incubated in PBS containing 3% normal goat serum and 0.3% Triton X-100 for 30 min at room temperature (RT). Brain sections were incubated for 48 hr at 4°C in a mixture of two primary antibodies: MK (1:100) in combination with mouse anti-GFAP (1:500; Sigma) or mouse OX-42 (1:200; Serotec, Oxford, UK). Sections were then incubated in a mixture of Alexa Fluor 488-conjugated goat anti-rabbit IgG (1:200; Molecular Probes) and Alexa Fluor 594-conjugated goat anti-mouse IgG (1:200; Molecular Probes) at RT for 2 hr in the dark. Processed sections were mounted on gelatin-coated slides, coversliped and examined under a Zeiss Axioplan-2 microscope.

### Quantitative analysis

Five coronal hippocampal sections (at the level of the injection site and spaced 60 μm from each other) were used for immunohistochemical analysis. All quantitative analyses were performed in a blind manner. To ensure consistency in tissue sampling, matched hippocampal sections (based on anatomical landmark) were always processed throughout the experiments. Digitized images of stained sections were acquired using a Zeiss Axioplan-2 microscope equipped with a DVC camera (Diagnostic Instruments, Sterling Heights, MI). These images were then analyzed using Northern Eclipse software (Empix Imaging, Mississauga, ON, Canada). Three microscopic fields within SP (stratum pyramidale) and Slm (stratum lacunosum moleculare) areas in CA3 were selected (magnification of ×40) in MK-stained sections. MK immunoreactivity was then measured and expressed as area density of MK (the fraction of the total given area occupied by MK-positive cells). Three microscopic fields (magnification at ×40) in CA1, CA3 and hilus region were counted for Nissl-stained undamaged neurons in each coronal brain section. Neurons are identified by dark nucleoli within lightly stained nuclei. GAD67-positive neurons were quantified in CA1 and CA3 subfields (strata oriens, radiatum, and pyramidale) and dentate gyrus layers (dentate hilus, granule cell layer, and molecular layer). These regions were established according to the atlas of Paxinos & Watson [36]. Number of Nissl- and GAD67-positive neurons was expressed as a percentage of the PBS-injected control sections.

### Statistical analysis

Data are presented as means SEM. The statistical significance was determined by one-way ANOVA and Student-Newman-Keuls test or Student's *t*-test using the StatView program (Abacus Concepts, Berkeley, CA). *p *values < 0.05 were considered to be statistically significant.

## Authors' contributions

YBK and JKR designed the study, carried out animal experiments and drafted the manuscript. HJL, IJL, and DP performed histology and histochemistry, and MCL participated in the design of the study and histological analysis. SUK conceived of the study, participated in its design and coordination, and drafted the manuscript. All authors read and approved the final manuscript.
